# Crimean-Congo Hemorrhagic Fever, Kosovo, 2013–2016

**DOI:** 10.3201/eid2502.171999

**Published:** 2019-02

**Authors:** Salih Ahmeti, Lindita Berisha, Bahrije Halili, Florim Ahmeti, Ronald von Possel, Corinna Thomé-Bolduan, Anett Michel, Simone Priesnitz, Emil C. Reisinger, Stephan Günther, Andreas Krüger, Kurtesh Sherifi, Xhevat Jakupi, Christoph J. Hemmer, Petra Emmerich

**Affiliations:** University Hasan Prishtina, Prishtina, Kosovo (S. Ahmeti);; University Clinical Center of Kosovo, Prishtina (L. Berisha, B. Halili);; National Institute of Public Health of Kosovo, Prishtina (F. Ahmeti, X. Jakupi);; Bernhard Nocht Institute of Tropical Medicine, Hamburg, Germany (R. von Possel, C. Thomé-Bolduan, S. Günther,; P. Emmerich); Bundeswehr Hospital, Hamburg (A. Michel, S. Priesnitz, A. Krüger);; University of Rostock, Rostock, Germany (E.C. Reisinger, C.J. Hemmer, P. Emmerich);; University Hasan Prishtina Faculty of Agriculture and Veterinary Science, Prishtina (K. Sherifi)

**Keywords:** CCHF, Crimean-Congo hemorrhagic fever, Kosovo, case-fatality rate, bleeding, somnolence, coma, viral load, LDH, ribavirin, viruses, lactate dehydrogenase

## Abstract

During 2013–2016, a total of 32 patients were treated for Crimean-Congo hemorrhagic fever in Prishtina, Kosovo; 11 died. In the 11 patients who died, findings included viral loads >1 × 10^8.5^/mL, lactate dehydrogenase >2,700 U/mL, bleeding, and impaired consciousness. Ribavirin therapy had no noticeable effect in this small patient sample.

Crimean-Congo hemorrhagic fever (CCHF), caused by an orthonairovirus of the *Nairoviridae* family, is usually transmitted by bites of *Hyalomma* sp. ticks. Case-fatality rates vary from 10% to 40%. Most cases are reported from the Balkans, the Middle East, and Asia (*1*), but CCHF virus is now also found in *Hyalomma* ticks in Spain, where human CCHF cases have occurred (*2*,*3*).

The earliest known case of CCHF in Kosovo was observed in 1954 (*4*). Since 1989, outbreaks have been seen every 4–5 years (*5*). CCHF is present in 50% of the Kosovar territory, especially the central and southwest, which is at low altitude, has a hot climate, and consists mainly of bush and farmland vegetation (*6*). The same study found a human CCHF seroprevalence in Kosovo of 24.3%, annual incidence of 0.49/100,000 population, and a case-fatality rate of 26.76% (for 1995–2009).

Incubation of CCHF may take up to 9 days. Signs and symptoms usually start 1–3 days after the tick bite and include sudden onset of fever, myalgia, neck stiffness, photophobia, vomiting, diarrhea, sore throat, agitation, and confusion; 2–4 days later, drowsiness and pain in the right upper quadrant of the abdomen may ensue. Other signs and symptoms include tachycardia, petechiae, and lymphadenopathy. Approximately 30% of patients die >5 days after disease onset or later, typically from bleeding or multiorgan failure (*5*).

We present a retrospective analysis of all 32 CCHF cases from the Infectious Diseases Hospital of Hasan Prishtina University (Prishtina, Kosovo) during May 2013–July 2016. We obtained a statement of approval from the Committee of Professional Ethics of the University Clinical Center of Kosovo (filed under no. 1555 on October 27, 2017).

## The Study

We analyzed records of 32 patients with CCHF and excerpted demographic patient data, case history, symptoms, clinical signs, laboratory results, and outcome. Viral loads had been determined by reverse transcription PCR (RT-PCR) using a RealStar CCHFV RT-PCR Kit version 1.0 (Altona Diagnostics, http://www.altona-diagnostics.com). We used SPSS software (IBM, http://www.ibm.com/analytics/spss-statistics-software) for statistical analysis; p values of <0.05 by χ^2^ test or logistical regression (2-sided where applicable) were considered significant.

**Figure 1:**
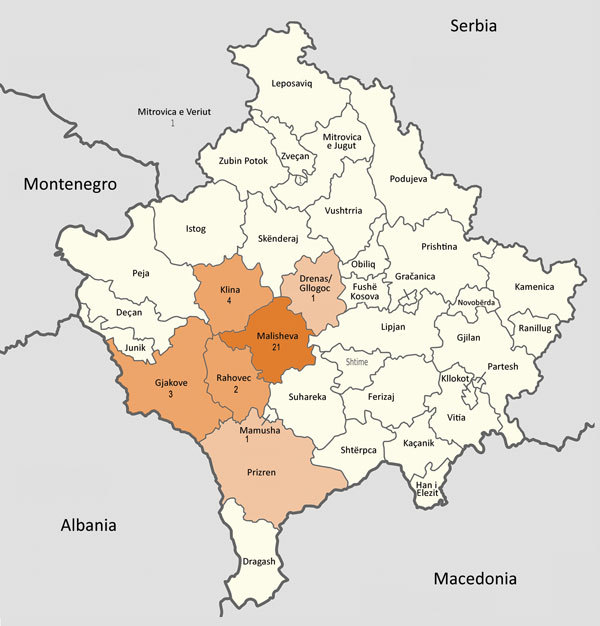
Municipalities (*komunë*) in Kosovo, showing number of patients with Crimean-Congo hemorrhagic fever in each municipality. Map was obtained from Wikimedia, where it is available to the public under the GNU Free Documentation License (*13*). The original map has no invariant elements; it has been modified to indicate patient locations.

**Figure 2:**
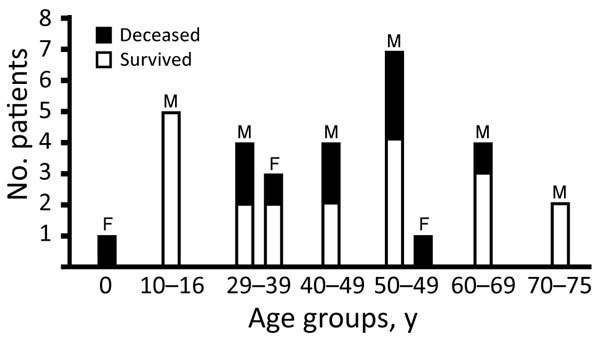
Crimean-Congo hemorrhagic fever cases and deaths by age group and sex, Kosovo, 2013–2016.

Of the 32 patients, 27 were male and 5 female; 21 patients were from the municipality of Malisheva (district of Prizren) and 9 from municipalities west of Malisheva (Figure 1). Median age was 40.5 years (range 0–75 years) (Figure 2). Most patients were exposed to ticks during farming.

Eleven patients died, including 1 vertically infected female newborn; 21 survived. The median duration between initial symptoms and hospital admission was 2.5 days (range 0–7 days). Neither χ^2^ testing nor regression analysis showed a relationship between the duration of symptoms at hospitalization (or at the start of ribavirin) and outcome.

Of the 6 patients who did not receive ribavirin, 2 died. Of the 26 patients who received intravenous ribavirin, 9 died. Ribavirin had no effect on outcome; however, the sample size was small. The median duration between the start of symptoms and administration of ribavirin was 3.5 days (range 1–12 days). Univariate regression (odds radio [OR] 13.3, 95% CI 1.3–134; p = 0.028) and χ^2^ testing (p = 0.055) suggested a possible association between starting ribavirin <5 days after disease onset and an increased probability of death. However, this effect was not significant after controlling for viral load (p = 0.19).

Signs of central nervous system (CNS) impairment (coma, somnolence, fasciculations) and bleeding showed the strongest interdependence with clinical outcome (Table 1). A weaker interdependence was shown for jaundice, diarrhea, and hiccups. Among the laboratory parameters, a viral load >1 × 10^8.5^ copies/mL, lactate dehydrogenase (LDH) >2,200 U/mL, and leukocyte count >7,700 cells/µL were associated with death. We saw a nonsignificant trend toward interdependence between platelet counts <50,000/µL and death (p = 0.057). We found no association with outcome for aspartate aminotransferase, alanine aminotransferase, or other laboratory parameters (data not shown).

**Table 1:** Interdependence between clinical and laboratory findings and outcome for patients with Crimean-Congo hemorrhagic fever, Kosovo, 2013–2016*

Finding	χ^2^	p value
Significant interdependence with outcome, with Yates correction		
Clinical findings		
Fasciculations	14.0	<0.001
Coma	13.2	<0.001
Somnolence	6.5	0.010
Hemoperitoneum	10.4	0.001
Bleeding gums	8.8	0.003
Hematemesis	7.7	0.005
Melena	5.7	0.017
Petechiae	5.7	0.017
Ecchymoses	5.5	0.019
Jaundice	6.2	0.013
Diarrhea	5.7	0.017
Hiccup	4.0	0.046
Laboratory findings		
Viral load >1 × 10^8.5^/mL	18.5	<0.001
LDH >2,200 U/mL	9.56	0.002
Leukocytes >7,700/µL	7.4	0.006
No interdependence with outcome, without Yates correction		
Clinical findings		
Vertigo	2.26	0.133
Hypertension	1.97	0.160
Anorexia	1.72	0.189
Nausea	1.60	0.205
Epistaxis	0.90	0.340
Vomiting	0.74	0.388
Joint pain	0.74	0.388
Sweating	0.23	0.631
Headache	0.13	0.722
Conjunctivitis	0.13	0.722
Muscular pain	0.08	0.773
Abdominal pain	0.05	0.830
Hypotension	0.03	0.864
Bradycardia	<0.01	0.968
Laboratory findings		
ALT >168 U/mL	1.54	0.215
AST >147 U/mL	1.47	0.225
CK >1,037 U/mL	0.79	0.373
Nonsignificant trend toward interdependence with outcome		
Platelets <50,000/µL	3.68	0.055
With Yates correction	3.63	0.057

*ALT, alanine aminotransferase; AST, aspartate aminotransferase; CK, creatine kinase; LDH, lactate dehydrogenase.

Of the clinical signs and symptoms, CNS impairment and bleeding predicted death. The death risk increased 35-fold (p = 0.003) in the presence of coma and 27-fold (p = 0.001) with somnolence; CNS impairment was present in all 11 patients who died. For fasciculations, we could not calculate an OR because no patient with fasciculations survived. Hemoperitoneum increased the death risk 16.6-fold (p = 0.004). If 4 of 5 bleeding signs (bleeding gums, hematemesis, melena, petechiae, ecchymoses) were present, the death risk increased 24-fold (p = 0.008). Diarrhea increased the death risk 7.2-fold (p = 0.023).

In multivariate analysis, the effects of bleeding, CNS involvement, and diarrhea on death risk were dependent on viral load and LDH levels. The effect of bleeding signs on death risk was independent of the effects of somnolence and of diarrhea. The effect of somnolence, but not of diarrhea, on death risk was independent of bleeding.

We saw no influence on death risk with other signs or symptoms (Table 2). Ribavirin therapy did not influence death risk (OR 1.059; p = 0.952) in the small sample size.

**Table 2:** Clinical and laboratory findings and their association with death in patients with Crimean-Congo hemorrhagic fever, Kosovo, 2013–2016*

Finding	OR for death if present (95% CI)	p value
Clinical findings significantly associated with death		
Coma	35.0 (3.32–369)	0.003
Somnolence	27.0 (3.80–192)	0.001
Fasciculations†	NA (25.2 [2.45–259])	NA (0.007)
Hemoperitoneum	16.6 (2.47–112)	0.004
Hematemesis	11.4 (1.74–74.7)	0.011
Bleeding gums	11.3 (2.04–63.1)	0.006
Ecchymoses	7.31 (1.25–42.8)	0.027
Melena	7.20 (1.31–39.6)	0.023
Petechiae	6.67 (1.31–34.0)	0.023
>4 of the 5 previous findings	24.0 (2.33–247)	0.008
Diarrhea	7.20 (1.31–39.6)	0.023
Jaundice†	NA (7.87 [0.71–87.3])	NA (0.093)
Clinical findings not significantly associated with death		
Vertigo	5.00 (0.53–47.3)	0.160
Female sex	3.56 (0.50–25.6)	0.206
Epistaxis	2.08 (0.46–9.51)	0.343
Sweats	2.00 (0.11–35.4)	0.636
Joint pain	1.92 (0.43–8.61)	0.391
Headache	1.31 (0.29–5.89)	0.723
Conjunctivitis	1.31 (0.29–5.89)	0.723
Abdominal pain	1.20 (0.23–6.34)	0.830
Tiredness	1.05 (0.08–13.1)	0.968
Bradycardia	1.05 (0.08–13.1)	0.968
Metrorrhagia	0.95 (0.08–11.1)	0.968
Hypotension	0.87 (0.19–4.03)	0.864
Muscular pain	0.75 (0.11–5.32)	0.774
Vomiting	0.52 (0.12–2.32)	0.391
Hyperemia	0.42 (0.09–1.86)	0.251
Anorexia	0.28 (0.04–2.01)	0.206
Nausea	0.25 (0.03–2.40)	0.230
Hiccup†	NA (4.67 [0.37–58.3])	NA (0.232)
Hypertension†	NA (2.10 [0.12–37.1])	NA (0.613)
Ribavirin not given	1.059 (0.162–6.94)	0.952
Laboratory findings significantly associated with death		
VL >1 × 10^8.5^	80.0 (6.3–1,011)	0.001
LDH >3,500 U/L†	NA (26.7 [2.24–317])	NA (0.009)
LDH >2,700 U/L	37.5 (2.77–507)	0.006
Leukocytes >7.7	15.8 (1.53–164)	0.020
Leukocytes >8.0†	NA (16.7 [1.62–172])	NA (0.018)
Platelets <50.000/µL	5.25 (1.07–25.8)	0.041
Laboratory findings not significantly associated with death		
ALT >168 U/L	3.20 (0.48–21.2)	0.228
AST >147 U/L	2.75 (0.52–14.4)	0.232
CK >1,037	1.95 (0.44–8.55)	0.376
IgG <2.5‡	NA (4.67 [0.45–48.3])	NA (0.196)
IgM <2.5‡	NA (4.67 [0.45–48.3])	NA (0.196)

*ALT, alanine aminotransferase; AST, aspartate aminotransferase; CK, creatine kinase; LDH, lactate dehydrogenase; NA, not applicable; OR, odds ratio; VL, viral load. †The OR could not be determined because there were no survivors with this symptom. The value in parentheses would have been obtained if 1 hypothetical survivor had this symptom or test result. ‡The OR could not be determined because there were no deaths among patients with this finding. The value in parentheses would have been obtained if 1 hypothetical fatal case had this test result.

The strongest predictor of death was a viral load >1 × 10^8.5^ viral copies/mL of serum (OR 80.0; p = 0.001), followed by an LDH >2,700 U/mL (OR 37.5; p = 0.006). The probability of death was also increased for leukocyte counts >7,700 cells/µL (OR 37.5; p = 0.006) and platelet counts <50,000/µL (OR 5.25; p = 0.041).

In multivariate analysis, viral loads >1 × 10^8.5^ copies/mL and LDH levels >2,700 U/mL independently increased the probability of death (Table 2). However, the effect of leukocyte counts >7,700 cells/µL and platelet counts <50,000/µL did not persist when controlled for viral loads or LDH levels. No other routine laboratory parameters, including hemoglobin, creatine kinase, aspartate aminotransferase, alanine aminotransferase, prothrombin time, partial thromboplastin time, CCHF virus IgG, and CCHF IgM virus, predicted the outcome in the patients we analyzed.

## Conclusions

Our results showed that the strongest clinical predictors of death from CCHF were bleeding and neurologic involvement. We saw somnolence or coma in all 11 fatal cases and bleeding in 9. In 2 patients, intracerebral bleeding could not be excluded because computed tomography or magnetic resonance imaging scans were not available. Therefore, the question whether all fatal neurologic complications in CCHF are caused by hemorrhage remains open.

Of laboratory parameters, the strongest predictors of death were viral load >1 × 10^8.5^ viral genome copies/mL (OR 80) and LDH level >2,700 U/mL (OR 37.5). The predictive value of viral load has been described in Kosovo and Turkey (*7*,*8*). High viral loads predicted high LDH levels, which in turn predicted complications and death. CCHF virus infection may induce organ failure by causing apoptosis of several cell types of endothelial and parenchymal origin (*9*). Other factors probably contribute to CCHF pathology also.

We saw a higher case-fatality rate in Kosovo (34%) than that reported in Turkey (11%) (*8*). Although the virus clade circulating in Kosovo was probably introduced from Turkey in the 1970s, it is possible that the high genetic viral diversity found in fatal CCHF cases in Kosovo increases CCHF pathogenicity (*10*).

Our study did not detect any benefit of intravenous ribavirin for CCHF; however, even a recent Cochrane meta-analysis has not answered the question whether ribavirin is beneficial in CCHF (*11*). A murine treatment study suggests that ribavirin is insufficiently effective even when given before disease onset (*12*). To improve the prognosis for CCHF, new antiviral substances that effectively curb virus replication are needed.
